# TMT-Based Proteomic Analysis of Continuous Cropping Response in *Codonopsis tangshen* Oliv.

**DOI:** 10.3390/life13030765

**Published:** 2023-03-13

**Authors:** Xiaogang Jiang, Wuxian Zhou, Hua Wang, Jinwen You, Wenlu Liu, Meide Zhang

**Affiliations:** 1 Key Laboratory of Biology and Cultivation of Herb Medicine, Ministry of Agriculture and Rural Affairs, Institute of Chinese Herbal Medicines, Hubei Academy of Agricultural Sciences, Enshi 445000, China; 2 Agricultural and Rural Bureau of Enshi Tujia and Miao Autonomous Prefecture, Enshi 445000, China

**Keywords:** *Codonopsis tangshen* Oliv., proteome, continuous cropping, biological process

## Abstract

The growth and development of *Codonopsis tangshen*, an important herb used in Chinese traditional medicine, have been seriously affected by continuous cropping obstacles. Therefore, understanding the molecular responses of *C. tangshen* to continuous cropping is imperative to improve its resistance to continuous cropping obstacles. Here, physiological and biochemical results showed that the levels of chlorophyll and malonaldehyde (MDA) were higher in the continuous cropping (LZ) group compared with those of the non-continuous cropping (FLZ) group, while superoxide dismutase (SOD) content was lower in the LZ group than in the FLZ group. Tandem mass tag (TMT)-based proteomic analysis was performed to investigate the response mechanism to continuous cropping obstacles in *C. tangshen*. A total of 70 differentially expressed proteins (DEPs) were significantly involved in relevant pathways, including photosynthesis, oxidative phosphorylation, ribosome activity, and secondary metabolites. The results suggest that these DEPs in *C. tangshen* might play a critical role in response to continuous cropping. These findings could provide scientific basis for improving *C. tangshen*’s resistance to continuous cropping obstacles.

## 1. Introduction

*Codonopsis tangshen* Oliv. is a perennial herbaceous plant that belongs to the family Campanulaceae and is used in Chinese traditional medicine [1]. The dry roots of *C. tangshen* are used for both medicine and food and have the effects of invigorating the spleen and lungs, nourishing blood, and generating fluid [2]. Medicinal ingredients, including polysaccharides, saponins, alkaloids, sesquiterpenes, glycosides, and polyphenolic polyacetylenes, have been identified in the roots of *C. tangshen* [3,4]. The plant is mainly cultivated in the Chongqing, Hubei, Sichuan, Guizhou, and Shanxi provinces of China [5]. *C. tangshen* can grow in half shade and half sunshine or on a shady slope at high altitude. There is a growing market demand for *C. tangshen*; besides its medicinal value, it is also edible and used as an ingredient in various products. However, continuous cropping can induce serious obstacles that inhibit the growth and development of *C. tangshen* and other plants. Previous studies have demonstrated that continuous cropping causes symptoms such as small, etiolated, and senescent leaves; thin and dry stems; poor lodging resistance; and greater vulnerability to diseases such as root rot and purple stripe feather disease [5]. Even more seriously, the disease can restrict the underground growth of *C. tangshen* and decrease the yield and quality of the root. Overall, continuous cropping obstacles are a serious problem for the growth and development of *C. tangshen*.

Other studies have revealed the influence of continuous cropping on cash crops and medicinal plants. For instance, in medicinal plants, soil nutrient imbalance, changes in microbial community structure, and the production of autotoxic substances were caused by continuous cropping obstacles [6,7,8]. A study on *Salvia miltiorrhiza* showed that continuous cropping resulted in significant declines in the diameter of main roots, weight of fresh roots, and total hydrophilic and lipophilic content [7]. A study on *Angelica sinensis* indicated that seedlings were significantly inhibited in growth, root yield, and quality following continuous cropping [8]. Replant disease in *Rehmannia glutinosa* caused by continuous cropping severely inhibited tuberous root formation, and fibrous roots were formed mostly underground, resulting in a significant decline in yield [9,10]. Additionally, research on *Panax notoginseng* revealed that continuous cropping made plants highly susceptible to fungal pathogens and reduced the yield and quality of tuber root [11]. Nevertheless, there are few studies about the effects of continuous cropping on the growth and development of *C. tangshen*.

Most previous studies have focused on the effect of continuous cropping on physiochemical properties, microbial community structure, and the growth and development of various crop species, but not its effects on plants at the molecular level. For instance, a study on *Andrographis paniculata* revealed that continuous cropping decreased the expression of genes related to terpenoid, phenylpropanoid, and flavonoid biosynthesis pathways [12]. A previous study on *C. tangshen* identified candidate genes involved in pathways such as tyrosine degradation I, glycogen synthesis, and phenylalanine and tyrosine catabolism [5]. Moreover, a study on *Rehmannia glutinosa* indicated that continuous cropping affected the gene expression related to metabolism, ROS generation, immune response, ER stress, and lignin synthesis [10]. These findings demonstrate that continuous cropping affected the expression of crucial genes involved in pathways, leading to disease, infection, the inhibition of growth and development, and a decrease in plant yield and quality.

In plants, protein turnover is the process of protein synthesis and degradation that is in dynamic equilibrium. However, internal and external stimuli can disturb the dynamic balance [13]. Proteome sequencing can be used to reveal insight into how plants respond to various stresses and regulate growth and development. Although several studies have investigated the mechanisms of continuous cropping in many plant species, how continuous cropping affected and has reprogrammed the physiological processes of *C. tangshen*, especially at the proteome level, remains largely unknown. In this study, proteome sequencing was adopted to determine *C. tangshen*’s molecular response mechanism to continuous cropping obstacles. Additionally, differentially expressed proteins (DEPs) and metabolic pathways significantly induced by continuous cropping were identified. These results will contribute to our understanding of the molecular response mechanism to continuous cropping obstacles and provide a basis for improving *C. tangshen*’s resistance to replanting obstacles.

## 2. Materials and Methods

### 2.1. Chemicals and Instrumentation

P-PER™ Plant Protein Extraction Reagent, a TMT10plex Isobaric Label Reagent Set, and a Pierce™ BCA Protein Assay Kit were purchased from Thermo Fisher Science (Waltham, MA, USA). Urea, triethylammonium bicarbonate buffer (1.0 M, pH 8.5 ± 0.1), tris (2-carboxyethyl) phosphine hydrochloride solution (0.5 M, pH 7.0), iodoacetamide (IAA), formic acid (FA), acetonitrile (ACN), and methanol were purchased from Sigma (St. Louis, MO, USA). Trypsin from bovine pancreas was purchased from Promega (Madison, WI, USA). Ultrapure water was prepared using a Millipore purification system (Billerica, MA, USA). We also used a Dionex Ultimate 3000 Nano LC system coupled with an Obitrap Q Exactive™ HF mass spectrometer (Thermo Fisher Scientific, Waltham, MA, USA) with an ESI nanospray source.

### 2.2. Plant Materials and Sample Collection

*C. tangshen* cultivated in Banqiao Town, Enshi City, Hubei Province was used as the experimental material. One field of *C. tangshen* was continuously cultivated using the continuous cropping (LZ) regime over 3 years, and one field had been previously cultivated with maize using a non-continuous cropping (FLZ) regime for several years. The two fields were adjacent to each other, with the same management measures for both treatments. The nitrogen application rate was 165.6 kg·hm^−2^, and m (N):m (P_2_O_5_):m (K_2_O) = 1:0.43:0.72. Urea, calcium magnesium phosphate fertilizer and potassium sulfate were used as nitrogen, phosphorus and potassium fertilizer, respectively. On 20 March 2021, the fertilizer of 50% nitrogen, 100% phosphorus and 100% potassium were applied to the soil, then the plots were fully plowed and the two fields were planted with fresh *C. tangshen* roots. The remaining 50% nitrogen fertilizer was applied at the seedling stage (20%) and flowering stage (30%) of *C. tangshen*. On 16 June 2021, young leaf samples were collected with three replicates for each treatment, and were used for proteome analysis. There were three biological replicates for each treatment. One portion set aside for sequence analyses was immediately frozen in liquid nitrogen, brought back to the laboratory, and stored in a −80 °C refrigerator until use. The remaining part was set aside for physiological and biochemical analyses and stored at 4 °C until use.

### 2.3. Measurement of Physiological and Biochemical Properties

Physio-chemical properties, including chlorophyll content, malonaldehyde and superoxide dismutase, were measured and analyzed according to methods developed in a previous study [14]. For chlorophyll content analysis, 0.1 g leaves were collected and immersed in 95% ethanol. The samples were stored in the dark for 48 h, then absorbance of the chlorophyll extract was measured at 665 and 649 nm using a spectrophotometer UV2600. The chlorophyll content was calculated according to the formula described in a previous study [15].

For MDA content determination, 0.3 g frozen leaves were used to extract the crude enzyme. Leaves were ground into powder in liquid nitrogen using a pre-chilled mortar and pestle (4 °C), homogenized with sodium phosphate buffer (PBS), pH 7.4, and centrifuged at 12,000 rpm for 20 min at 4 °C. The supernatant was used to measure the MDA content. A total of 1 mL extract was mixed with a 2 mL reaction solution containing 20% (*v*/*v*) trichloroacetic acid and 0.5% (*v*/*v*) thiobarbituric acid. The mixture was boiled in a water bath for 30 min, cooled to room temperature, and centrifuged at 12,000× *g* for 10 min at 20 °C. The absorbance was read at 532 and 600 nm with a spectrophotometer (UV2600, UNIC, Shanghai, China). The MDA content was calculated using the following formula: MDA (nmol g^−1^ FW) = [(A532 − A600) × V × 1000/ε] × W, where A600 and A532 are the absorbances at 600 and 532 nm, respectively, ε is the specific extinction coefficient (155 mM cm^−1^), V is the volume of crushing medium and W is the fresh weight of leaves (g).

The physiological and biochemical properties traits analysis was performed with three biological replicates, and one-way ANOVA was used to analyze differences between the FLZ and LZ group.

### 2.4. Protein Extraction and Digestion

The leaf proteins were extracted using P-PER™ Plant Protein Extraction Reagent. Briefly, 1.75 mL of P-PER™ Plant Protein Extraction Reagent was added into 1 g of plant tissue. The samples were then centrifugated into partitions of organic and aqueous phases at 5000× *g* for 5 min. The protein concentration was measured using the Pierce™ BCA Protein Assay Kit (Thermo Fisher Scientific, Waltham, MA, USA). We diluted 100 μg of protein extracts with 100 mM of TEAB for a final volume of 100 μL. Each sample tube was reduced by 10 mM TCEP at 56 °C for 1 h and alkylated by 20 mM of room temperature IAA in the dark for 1 h. We then added 180 μL of pre-chilled (−20 °C) acetone into the sample and froze it at −20 °C overnight. The mixtures were clarified by centrifugation at 8000× *g* for 10 min (4 °C). The acetone was carefully removed without disturbing the white pellet and the pellet was dried for 2–3 min. 100 μg of acetone-precipitated protein pellets was resuspended with 100 μL of 50 mM TEAB. Finally, 2 μg free trypsin was added into the protein solution and the solution was incubated at 37 °C overnight.

### 2.5. Labelling and Peptide Fractionation

Immediately before use, the TMT (Thermo Fisher Scientific, Waltham, MA, USA) Label Reagent was equilibrated to room temperature. We added 41 μL of anhydrous acetonitrile to each tube. The reagent was dissolved for 5 min with occasional vortexing. The samples were briefly centrifugated to gather the solution, and the reaction was incubated for 1 h at room temperature. We added 8 μL of 5% hydroxylamine to the samples and incubated them for 15 min to quench the reaction. Three LZ samples were labeled with 129 C-tag, 130 C-tag, 131-tag, respectively, while the FLZ samples were labeled with 127 N-tag, 128 N-tag, 129 C-tag, respectively. The labeled samples were mixed in equal amounts in new microcentrifuge tubes. Subsequently, mixed samples were divided into eight fractions using the Pierce™ High pH Reversed-Phase Peptide Fractionation Kit.

### 2.6. Liquid Chromatography–Mass Spectrometry (LC-MS/MS) Analysis and Database Search

The nano LC-MS/MS was subcontracted to Beijing Biotech-Pack Scientific (Beijing, China). The LC-MS/MS processing was carried out using the Dionex Ultimate 3000 Nano LC system (Thermo Fisher Scientific, Waltham, MA, USA) coupled with a Q-Exactive Mass Spectrometer and an ESI nanospray source. 0.1% FA in water and ACN were provided for mobile phase A and B, respectively. The total flow rate was 600 nL/min and a 120 min gradient was set as follows: 4–10% B, 5 min; 10–22% B, 80 min; 22–40% B, 25 min; 40–95% B, 5 min; and 95–95% B, 5 min. The spray voltage was set at 2.0 kV. All MS and MS/MS spectra were acquired in data-dependent acquisition mode and the full mass scan acquired 300–1400 m/z with a resolution of 120,000.

Raw MS data were analyzed and searched against the *Arabidopsis thaliana* protein database based on the species of samples using Proteome Discover 2.1. Trypsin was selected as the enzyme and two missed cleavages were allowed. Cysteine residues were set as the static modification and the oxidation of methionine was set as the variable modification. The mass tolerance of the precursor was 15 ppm and the peptide false discovery rate (FDR) was controlled ≤1%.

### 2.7. Protein Quantification and Bioinformatics Analysis

To identify the proteins that were significantly differentially expressed across the two groups of samples, the threshold values for the down-regulated and up-regulated proteins were 0.67-fold and 1.50-fold, respectively, with *p*-values less than 0.05. The identified proteins were annotated using the UniProt knowledge base (Swissprot/TrEMBL, http://www.uniprot.org/ (accessed on 21 March 2022)). The multi-omics data analysis tool, OmicsBean (http://www.omicsbean.cn/ (accessed on 21 March 2022))., was used to analyze the obtained proteomics data, in which distributions in biological process (BP), cellular components (CCs), and molecular functions (MF) were assigned to each protein based on Gene Ontology (GO) categories. Kyoto Encyclopedia of Genes and Genomes (KEGG) pathway analysis (http://www.genome.ad.jpkegg/pathway.html (accessed on 21 March 2022)) was performed to enrich high-level functions in the defined biological systems. Protein-protein interaction (PPI) analysis was performed using Cytoscape software version 3.9.1, with a confidence cutoff of 400. Interactions with larger confident scores were indicated with solid lines between genes/proteins, and others were shown with dashed lines.

### 2.8. Quantitative Real-Time Polymerase Chain Reaction (qRT-PCR) Analysis

Real-time quantitative RT-PCR was employed to validate the proteome data. Total RNA was extracted from frozen leaves using the Trizol reagent (Invitrogen, Carlsbad, CA, USA) following the manufacturer’s procedure. The quality of RNA extract was checked by 1% agarose gels and the NanoPhotometer^®^ spectrophotometer (IMPLEN, Westlake Village, CA, USA). A total of 2 µg of RNA extract was used to prepare cDNA for each sample using RevertAid First Strand cDNA Synthesis Kit (Invitrogen, Carlsbad, CA, USA). The PCR reaction was performed using a total of 20 µL, consisting of 10 µL of SYBR mix, 0.5 µL of each primer, 2 µL of cDNA, and 7µL dd water. PCR reaction was carried out on the ABI StepOne Plus Real Time PCR system (Applied Biosystems, Foster City, CA, USA). Melting curve analysis was used to check the specificity of the amplified PCR product. The *glyceraldehyde-3-phosphate dehydrogenase* (*GAPDH*) gene was used as the reference gene. The primers used in this study are listed in Table S1. The 2^−ΔΔCT^ method was used to determine the relative expression of genes [16]. One-way ANOVA was applied to analyze the statistical differences between different groups.

## 3. Results

### 3.1. Physiological and Biochemical Properties

**Figure 1:**
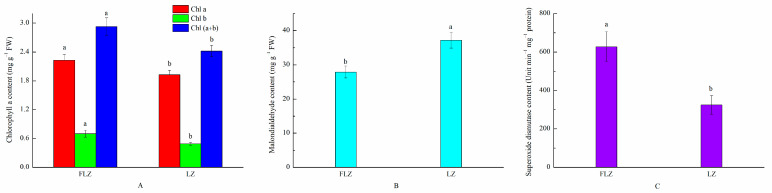
Content of chlorophyll (**A**), Malondialdehyde (**B**), and Superoxide dismutase (**C**) in FLZ and LZ *C. tangshen* leaves. The different letters indicate significant difference at the 0.05 probability level.

The physiological and biochemical traits were analyzed between the FLZ and LZ groups (Figure 1). The results indicated that the Chl a, Chl b, and total Chl contents of LZ were significantly lower than those of FLZ. Additionally, compared with FLZ, LZ showed significantly higher MDA content. However, SOD content was lower in LZ than in FLZ. The raw data for the physio-chemical properties are shown in Table S2.

### 3.2. Mass Spectrometry Identification

TMT-labeled quantitative proteomics analysis is a relative quantification method. In this analysis, we performed mass spectrometry detection on the proteome between FLZ and LZ. A total of 528 high-quality peptides were detected using various protein databases. The mass spectrometry proteomics data were deposited to the ProteomeXchange Consortium (http://proteomecentral.proteomexchange.org (accessed on 18 July 2022)) via the iProX partner repository with the dataset identifier PXD035371. After the merging process, we obtained 161 non-redundant proteins (Table S3). A total of 70 differentially expressed proteins, including 27 up-regulated and 43 down-regulated proteins were identified in continuous cropping (LZ) compared to non-continuous cropping (FLZ) (Table S4). In order to ensure the reliability of the identification results, we did a peptide matching error distribution analysis (Figure S1) to prove that the identification results were reliable.

### 3.3. Gene Ontology (GO) Analysis of DEPs in Response to Continuous Cropping

**Figure 2:**
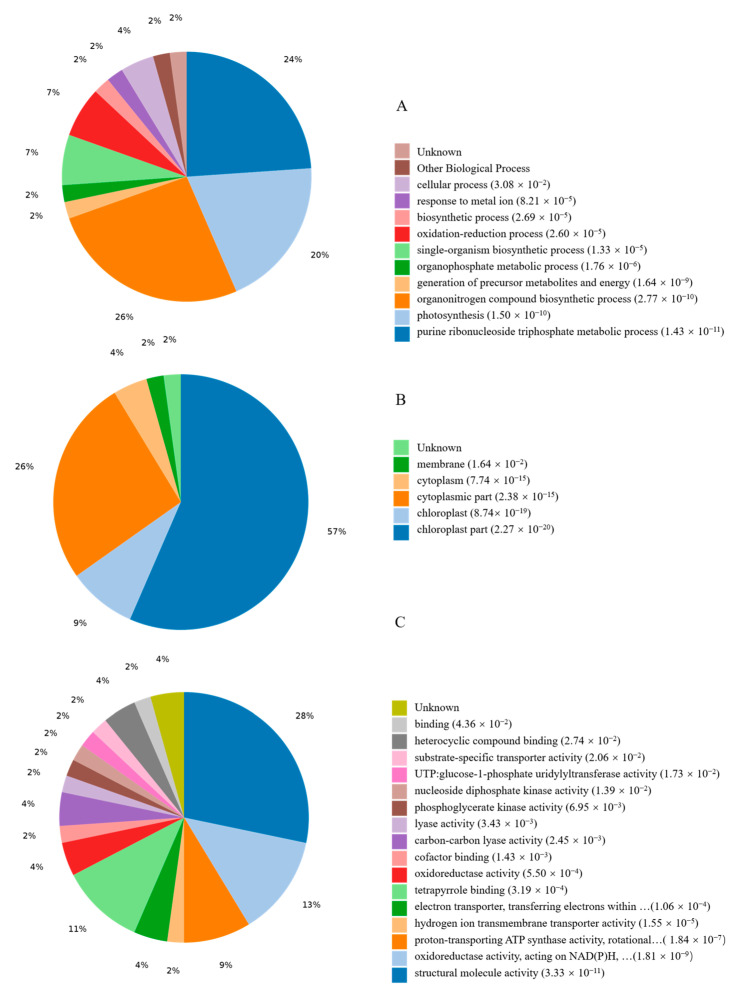
The statistics results of enriched Go terms. DEPs were classified into three categories: (**A**) biological process, (**B**) cell component, and (**C**) molecular function.

GO analysis was conducted to reveal the representation of proteins in response to continuous cropping based on *p*-values (FDR) < 0.05. Seventy DEPs were subdivided into categories of biological processes (BP), molecular functions (MF), and cell compositions (CC) (Figure S2). The Venn diagram in Figure S2 shows the number and overlap of proteins involved in the interactions of the three categories. In addition, enrichment for all GO terms indicated that 10 terms were significantly enriched in biological processes, such as the purine ribonucleoside triphosphate metabolic process, purine ribonucleotide metabolic process, purine ribonucleoside metabolic process, ribonucleoside triphosphate metabolic process, and purine nucleoside triphosphate metabolic process (Figure 2). In the cell components, 10 terms were largely enriched, including chloroplast, chloroplast thylakoid membrane, chloroplast thylakoid, plastid, and chloroplast part (Figure 2). In molecular function, the most enriched 10 terms included ATPase activity, proton-transporting ATP synthesis activity, NADH dehydrogenase (ubiquinone) activity, NADH dehydrogenase (quinone) activity, and rRNA binding (Figure 2).

### 3.4. KEGG Pathway Analysis of Responsive DEPs

**Figure 3:**
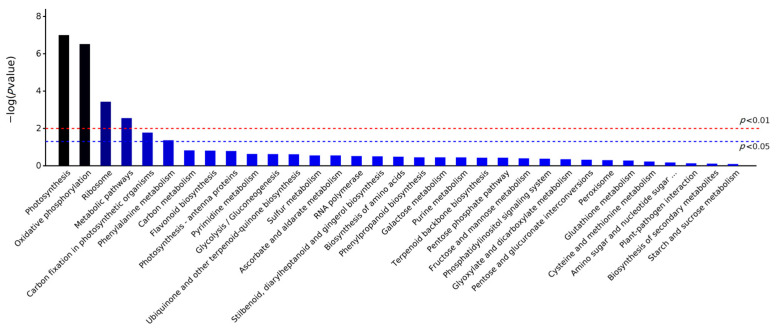
Significantly enriched pathway of DEPs in LZ *C. tangshen* compared with FLZ plants.

KEGG is a manually curated pathway database. According to the KEGG database, pathways are clustered into the following sub-categories, (A) Metabolism, (B) Genetic Information Processing, (C) Environmental Information Processing, (D) Cellular Processes, (E) Organismal Systems, and (F) Human Diseases. Enrichment analysis of KEGG pathways was performed with the same hypergeometric algorithm used in the GO enrichment analysis. To determine which pathway was active in the sample of continuous cropping plants, 70 DEPs were clustered into 33 KEGG pathways (Figure S3), and these proteins were significantly involved in pathways such as metabolic pathways, photosynthesis, oxidative phosphorylation, carbon fixation in photosynthetic organisms, phenylalanine metabolism, and ribosome based on *p*-values (FDR) < 0.05 (Figure 3). 

In metabolic pathways, proteins related to global and overview maps, energy metabolism, and amino acid metabolism were more abundant in LZ *C. tangshen*. These differentially expressed proteins may play an important role via these signaling pathways. All the enriched proteins in the main pathway are shown in Table 1. 

### 3.5. PPI Network Analysis

**Figure 4:**
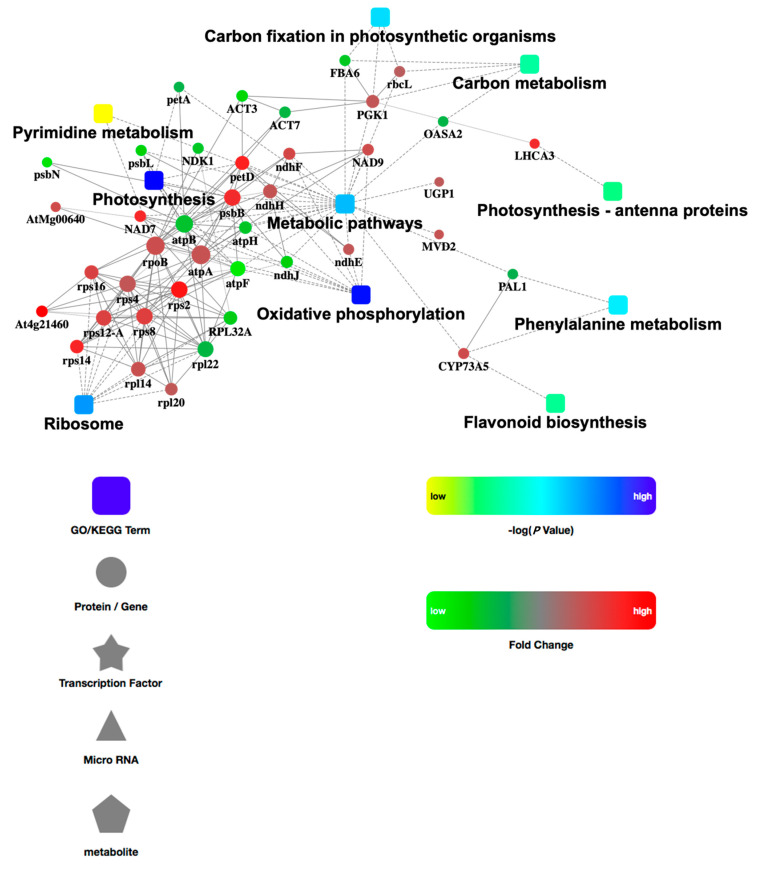
Protein-protein interaction network among the identified DEPs.

In order to further examine the comprehensive information obtained from the identified DEP data, we analyzed the PPI network. The network model was generated using the Cytoscape Web application based on four levels of functional analysis: fold-change of gene/protein expression, PPI, KEGG pathway enrichment, and biological process enrichment. A merged network is shown in Figure 4. In the network, the up-regulated proteins are indicated with red circle nodes and the down-regulated proteins are indicated with green circle nodes. A total of 40 DEPs, made up of 15 up-regulated and 25 down-regulated proteins, were involved in PPI. These proteins were related to 10 functional pathways: metabolic pathways, ribosome, oxidative phosphorylation, photosynthesis, carbon fixation in photosynthetic organisms, carbon metabolism, phenylalanine metabolism, pyrimidine metabolism, photosynthesis, and flavonoid biosynthesis.

### 3.6. Validation of the DEPs by qRT-PCR

**Figure 5:**
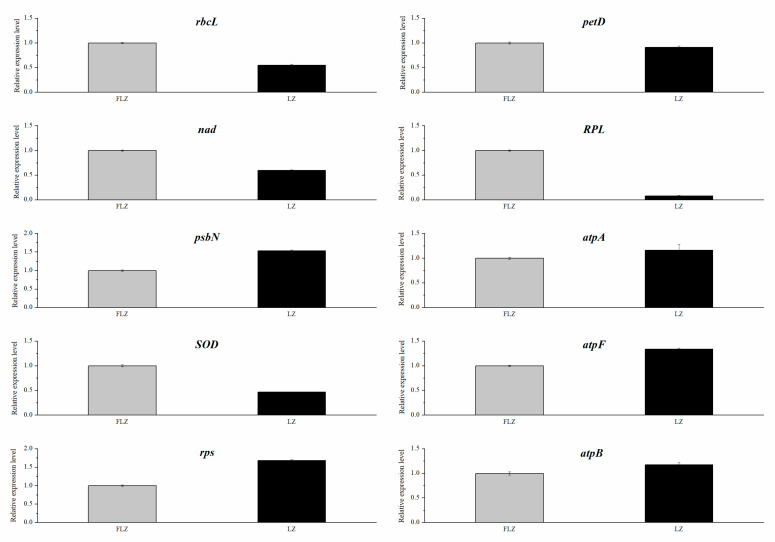
The qRT-PCR analysis of 10 genes in the leaves of LZ and FLZ plants.

The proteome results were validated by analyzing the expression of ten genes related to stress responses using qRT-PCR. The results indicated that the expression level of the five up-regulated genes (*RPS*, *psbN*, *atpA*, *atpB*, and *atpF*) was significantly higher in the LZ group than in the FLZ group. However, as shown in Figure 5, LZ greatly inhibited the expression of the five downregulated genes (*rbcL*, *nad*, *SOD*, *pet D*, *RPL*), compared with the FLZ. In summary, the expression profiles of these genes from the qRT-PCR were consistent with that from the proteome, indicating that the proteome data were reliable and accurate.

## 4. Discussion

### 4.1. Physiological and Biochemical Response in Continuously Cropped C. tangshen 

Continuous cropping obstacle is a severe abiotic factor limiting plants’ growth and development. Low levels of leaf chlorophyll can limit photosynthetic efficiency, consequently inhibiting the growth of plants. Previous studies have revealed that continuous cropping could induce lower chlorophyll content in plants such as potato and *Rehmannia* [17,18]. Similarly, our results demonstrated that chlorophyll (Chl a, Chl b) content declined after continuous cropping. One study showed the level of antioxidant enzymes SOD and the stress marker malonaldehyde (MDA) in the cells reflected the degree of oxidative damage [16,19]. In the present study, LZ significantly reduced the SOD activity and enhanced the MDA content, suggesting that the reduced SOD activity causing oxidative damage was indicated by increased malonaldehyde (MDA) contents. To investigate the molecular mechanisms of continuous cropping response in *C. tangshen,* we also performed proteome analysis. The results indicated one SOD protein was significantly differentially expressed between LZ and FLZ, which was consistent with SOD enzyme activity change (Table S4). The consistency demonstrated that the physiological activities of SOD enzyme in plants were disturbed in LZ plants. Similar result was reported in *Rehmannia glutinosa* under continuous cropping conditions [10]. Overall, the physiological and biochemical response consequently inhibited the growth and development of *C. tangshen*.

### 4.2. Proteomic Patterns of C. tangshen in Response to Continuous Cropping

Continuous cropping obstacles have seriously inhibited the growth and development of plants and have been a pervasive problem for agricultural production [6,20]. The effect of continuous cropping on the growth and development of *C. tanshen* is systematic and complicated. A previous study utilized transcriptome analysis to identify candidate genes related to biological processes and significant pathways, including the hormone signaling pathway, photosynthesis biological process, and important transcription factors (TFs) in response to continuous cropping in *C. tanshen* [5]. However, that study was conducted only at the transcriptional level, considering that some genes might occur in post-transcriptional events or could not be translated into protein products [21,22]. In this study, proteome-sequencing was applied to further validate and complement the mechanism that previous studies have elucidated, and 70 significantly differentially expressed proteins (27 up-regulated and 43 down-regulated proteins) were found in continuously-cropped *C. tanshen* compared to normal-growth plants. The results indicated these proteins related to dynamic biologic activities showed synergetic effect in response to continuous cropping in *C. tanshen.*

### 4.3. DEPs Related to Photosynthesis Pathway

**Figure 6:**
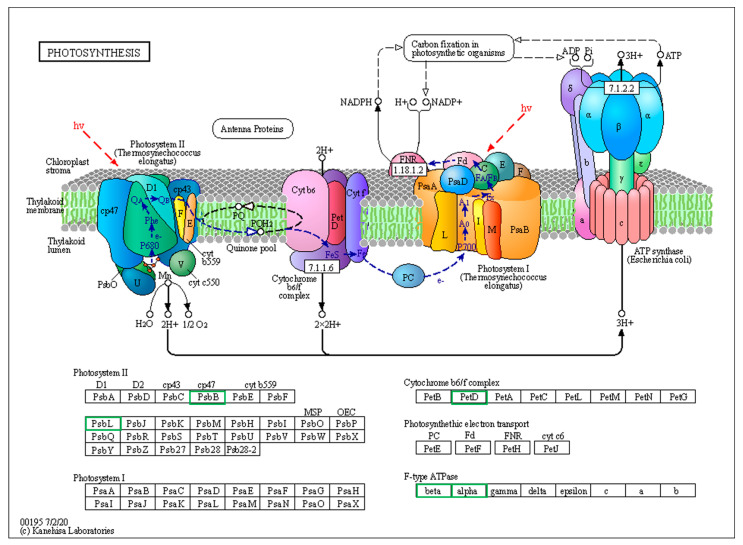
Differentially expressed proteins (DEPs) involved in photosynthesis pathway in LZ vs. FLZ treatment. Green frame denoted down-regulated protein. Black frame denoted proteins without differential expression.

Photoreaction and the Calvin cycle are critical reaction pathways of photosynthesis. Further analysis identified 28 differentially expressed proteins involved in photoreaction and the Calvin cycle. The significantly expressed DEPs relating to photoreaction included ATP synthase, photosystem II protein (psbB), chlorophyll a-b binding protein (Lhc), and cytochrome b6-f complex subunit (Figure 6). Of the 11 photoreaction-related proteins identified, nine were down-regulated, including six ATP synthase subunit, one photosystem II protein, one chlorophyll a-b binding protein, and one cytochrome b6-f complex subunit, which basically covered the whole process of photoreaction. The F-ATP synthase complex, which consists of Fo and F1 sectors, plays a pivotal role in energy-converting membranes and energetic demand to counteract abiotic stress [23]. Since ATP produced by photoreaction is required for carbon dioxide fixation in the Calvin pathway [24], down-regulated ATP synthase consequently caused limited energy provision utilized during carbon dioxide fixation.

PsbB protein is a core component of the multisubunit protein complex PSII embedded in the thylakoid membranes that mainly perform photoreaction [25]. Compared to the FLZ group, one psbB protein was down-regulated in the LZ group. This suggested that the composition of the PSII complex was influenced, which further inhibited the photoreaction efficiency and biomass accumulation under continuous cropping conditions.

Additionally, the expression of chlorophyll a-b binding protein (Lhc) decreased in the LZ treatment compared to FLZ. Other studies have demonstrated that Lhc are important components of PSII that are involved in light capture, energy transfer, and oxidative stress [26,27]. Based on our results, we can infer that down-regulated Lhc proteins not only limited light absorbtion for photoreaction, but also decreased the stress tolerance of *C. tanshen* after continuous cropping.

Moreover, 17 DEPs involved in the Calvin cycle were also identified. Among these proteins, 14 Ribulose bisphosphate carboxylase/oxygenase were down-regulated. Ribulose 1,5-bisphosphate carboxylase/oxygenase (Rubisco) was involved in the process of carbon fixation and transformation of atmospheric CO_2_ into biomass [28]. It was reported that the reduction of Rubisco protein content and CO_2_ assimilation were strongly related to a significant reduction in grain yield [29]. Therefore, these results suggested that the limited energy produced by photoreaction induced less CO_2_ fixation and finally decreased the photosynthetic output of *C. tanshen* after continuous cropping.

### 4.4. DEPs Associated with Oxidative Phosphorylation

NADH dehydrogenase (NADH DH), located in the chloroplast thylakoid and mitochondria, involves the cyclic electron transport to heat production and substrate ATP synthesis, and provides energy for physiological activity in response to photoinhibition [30,31]. In addition, a study showed that NADH dehydrogenase and its involved oxidative phosphorylation were closely associated with ROS production and contributed to oxidative stress in plants [32,33]. In our study, two NADH dehydrogenase subunits were down-regulated in LZ, while all seven ATP synthesis subunits were significantly up-regulated, which indicated that the decreased NADH DH and increased ATP synthesis in oxidative phosphorylation activated the ROS production in a continuous cropping system, further affecting the growth and development of *C. tanshen*.

### 4.5. DEPs Related to Ribosome Activity

In our study, six ribosomal proteins (rps2, rps8, rps12, rps16, rps14, and rpl20) were down-regulated after continuous cropping. Studies have indicated that the loss of ribosomal proteins probably reduced ribosome production and protein synthesis, thus leading to reduced growth [34]. Additional studies on *Arabidophis* have demonstrated that ribosomal-protein-defective mutants can cause growth defects and abnormal leaf development [35,36]. These results suggested that the low expression of ribosomal proteins might have a close relationship with symptoms such as smaller leaves, chlorosis, senescence, shorter and thinner stems, and lower lodging resistance for *C. tanshen* grown in the continuously cropped land [5].

### 4.6. DEPs Related to Secondary Metabolites

Diphosphomevalonate decarboxylase is involved in the synthesis of isoprenoids, the precursor to terpenoids [37]. Previous studies have revealed that terpenoids, a category of phenolic allelochemicals, could easily cause soil sickness in plants, resulting in a decline in crop yield [38]. In this study, diphosphomevalonate decarboxylase (MVD) protein was up-regulated, which was consistent with a different study on *Rehmannia glutinosa* [10]. This result suggested that continuous cropping greatly promoted the terpenoid output of *C. tanshen*, which led to the decrease in resistance to pathogens and soil sickness and inhibited the growth and development of *C. tanshen.*

In our study, cytosolic ascorbate peroxidase (cAPX) was down-regulated in continuously-cropped *C. tanshen*. cAPX involved in the ascorbate (AsA)-glutathione (GSH) cycle plays a critical role in the antioxidant defense system, nitrogen deficiency stress, and salt and heat tolerances in plants [39,40,41]. The above results indicate that the decreasing ascorbate (AsA)-glutathione (GSH) would lead to the limited anti-abiotic ability of *C. tanshen* cultivated under a continuous cropping system. Moreover, we found phenylalanine ammonia-lyase protein (PAL) was highly expressed in continuously cropped *C. tanshen*. Similarly, the highly expressed PALs were identified in replanted *Rehmannia glutinosa* [42]. PAL plays a critical role in lignin synthesis and lignin deposition, which could strengthen the cell wall to resist environmental stress [43]. Based on the results, we infer that PAL could only moderately alleviate the damage of continuous cropping obstacles to *C. tanshen*.

## 5. Conclusions

In our study, TMT-based proteomic sequencing was first utilized to reveal *C. tangshen*’s molecular response to continuous cropping obstacles. Our results indicated that identified DEPs between the FLZ and LZ groups were involved in pathways including photosynthesis, oxidative phosphorylation, ribosome activity, and secondary metabolites. These responsive proteins play a pivotal role in *C. tangshen*’s response to continuous cropping. In addition, proteome profiling results were consistent with the physio-biochemical traits, e.g., chlorophyll and SOD activity. Further analysis of these DEPs will be valuable for improving *C. tangshen* Oliv’s tolerance to continuous cropping obstacles in field cultivation.

## Figures and Tables

**Table 1:** Detailed information of DEPs involved in significantly enriched pathways.

Accession Number	Pathway and Annotation	Mean Ratio	MW [kDa]	pI	Score Mascot
	Photosynthesis pathway				
A0A1U7AF74	Cytochrome b6-f complex subunit 4	0.322	18.9	6.04	58
A0A2D1PBS6	Photosystem II 47 kDa protein	0.368	56.1	6.90	665
A0A291F2T6	Photosystem II reaction center protein L	2.211	4.5	4.50	112
A0A1L6BTC9	ATP synthase subunit alpha	0.574	54.9	6.34	671
A0A291F242	ATP synthase subunit alpha	0.571	54.9	6.04	600
B9U4M6	ATP synthase subunit beta	0.104	50.5	5.36	1120
B9U4I8	ATP synthase subunit beta	0.479	50.7	6.06	1198
H6VP95	ATP synthase subunit beta	0.369	47.1	5.83	1173
K9UVR2	Chlorophyll a-b binding protein	0.368	21.3	5.55	461
A0A288W750	ATPase subunit 4	0.524	21.9	9.69	26
A0A075DZC7	Protein PsbN	2.621	4.7	4.28	31
	Carbon fixation in photosynthetic organisms				
T1WIC8	Ribulose bisphosphate carboxylase large chain	1.950	26.9	6.01	842
A0A023Q1G8	Ribulose bisphosphate carboxylase large chain	0.488	41.1	7.61	2141
A0A023Q3H2	Ribulose bisphosphate carboxylase large chain	0.629	41.7	7.21	2563
U6BNI3	Ribulose bisphosphate carboxylase large chain	0.203	37.9	8.03	2216
C7AQR5	Ribulose bisphosphate carboxylase large chain	0.615	23.2	7.80	553
A0A023Q2I2	Ribulose bisphosphate carboxylase large chain	0.420	28.6	9.13	1361
A0A023Q1M8	Ribulose bisphosphate carboxylase large chain	0.175	36.2	8.53	2133
C7AQT3	Ribulose bisphosphate carboxylase large chain	2.386	50.1	7.02	1668
H6VPA6	Ribulose bisphosphate carboxylase large chain	0.580	48.6	6.80	1569
Q37184	Ribulose bisphosphate carboxylase large chain	0.639	52.5	6.70	2548
C3S8M7	Ribulose bisphosphate carboxylase large chain	0.538	50.7	6.68	1821
G8D4X5	Ribulose bisphosphate carboxylase large chain	1.511	22.4	6.05	607
C3S8S0	Ribulose bisphosphate carboxylase large chain	0.374	50.2	6.71	1209
A2VAL3	Ribulose bisphosphate carboxylase large chain	0.426	51.7	6.95	736
A0A1L6BRS7	Ribulose bisphosphate carboxylase large chain	0.628	52.6	6.70	1522
A6XBE1	Ribulose-1,5-bisphosphate carboxylase/oxygenase	0.319	20.3	7.27	1105
A0A1D6XPB7	Ribulose-1,5-bisphosphate carboxylase/oxygenase	0.632	12.0	7.34	230
	Ribosome				
A0A240FG30	30S ribosomal protein S14	0.344	11.8	11.53	28
A0A220D819	50S ribosomal protein L20	0.610	15.0	11.27	26
A0A2D1PBT7	Chloroplast 30S ribosomal protein S4	0.594	23.3	10.51	131
A0A1Z2QRI8	Ribosomal protein L14	0.556	13.5	9.19	176
A0A291F5L8	Ribosomal protein L22	1.687	32.5	9.54	75
K9UUF3	Ribosomal protein RPL32e	2.096	10.6	10.29	26
A0A1L6BUB7	Ribosomal protein S12	0.469	14.1	11.46	45
A0A2D1PBQ4	Ribosomal protein S16	0.475	10.5	10.11	24
A0A2D1PBU9	Ribosomal protein S2	0.287	26.9	9.69	45
A0A291F0G4	Ribosomal protein S8	0.451	15.9	10.87	94
	Oxidative phosphorylation				
A0A2D1PBY9	NAD(P)H-quinone oxidoreductase chain 5	0.504	84.9	9.10	21
A0A2D1PBT2	NAD(P)H-quinone oxidoreductase chain J	2.160	18.8	6.79	37
A0A240FG85	NAD(P)H-quinone oxidoreductase subunit 4L	0.593	11.2	9.72	33
A0A088PYE4	NAD(P)H-quinone oxidoreductase subunit H	0.583	45.5	5.87	48
A0A288W7F2	NADH dehydrogenase subunit 7	0.354	44.2	7.05	69
A0A0K1ZFQ7	NADH dehydrogenase subunit 9	0.531	22.5	7.46	0
A0A240FG45	Cytochrome f	1.622	35.1	8.97	822
A0A1L6BSB5	ATP synthase CF0 subunit I	2.819	21.0	6.02	0
H6VP90	ATP synthase subunit beta (Fragment)	2.091	47.8	5.19	1609
A0A1Z2QTG2	ATP synthase subunit beta	2.267	53.7	5.58	1479
B3SU35	ATP synthase subunit beta, chloroplastic	1.870	53.7	5.72	1461
A0A220D850	ATP synthase subunit c, chloroplastic	1.969	11.0	4.64	31
B9U4M3	ATP synthase subunit beta (Fragment)	1.907	49.5	5.08	1707
A0A0K1Z6V1	ATP synthase subunit alpha	4.268	54.9	5.91	560
	Secondary metabolites				
V9PEM0	Diphosphomevalonate decarboxylase	0.578	46.0	6.38	19
J7EQD9	Phenylalanine ammonia-lyase	1.557	77.1	6.10	264
Q2WFK7	Cytosolic ascorbate peroxidase	1.620	27.9	6.00	242

## Data Availability

The mass spectrometry proteomics data were deposited with the ProteomeXchange Consortium (http://proteomecentral.proteomexchange.org, accessed on 18 July 2022) via the iProX partner repository with the dataset identifier PXD035371.

## Supplementary Materials

The following supporting information can be downloaded at: https://www.mdpi.com/article/10.3390/life13030765/s1, Figure S1: Peptide matching error distribution; Figure S2: Venn diagram for DEPs between FLZ and LZ group; Figure S3: KEGG enrichment of DEPs between FLZ and LZ group; Table S1: The qRT-PCR primers used in this study title; Table S2: The raw data for the physio-chemical properties; Table S3: Detailed information of annotated proteins; Table S4: Annotation of DEPs between FLZ and LZ group.
