# Supplementary figures and images for: TMT-Based Proteomic Analysis of Continuous Cropping Response in Codonopsis tangshen Oliv
Source: Life (Basel). 2023 Mar 13;13(3):765. doi: 10.3390/life13030765 (PMC10052164; doi:10.3390/life13030765)

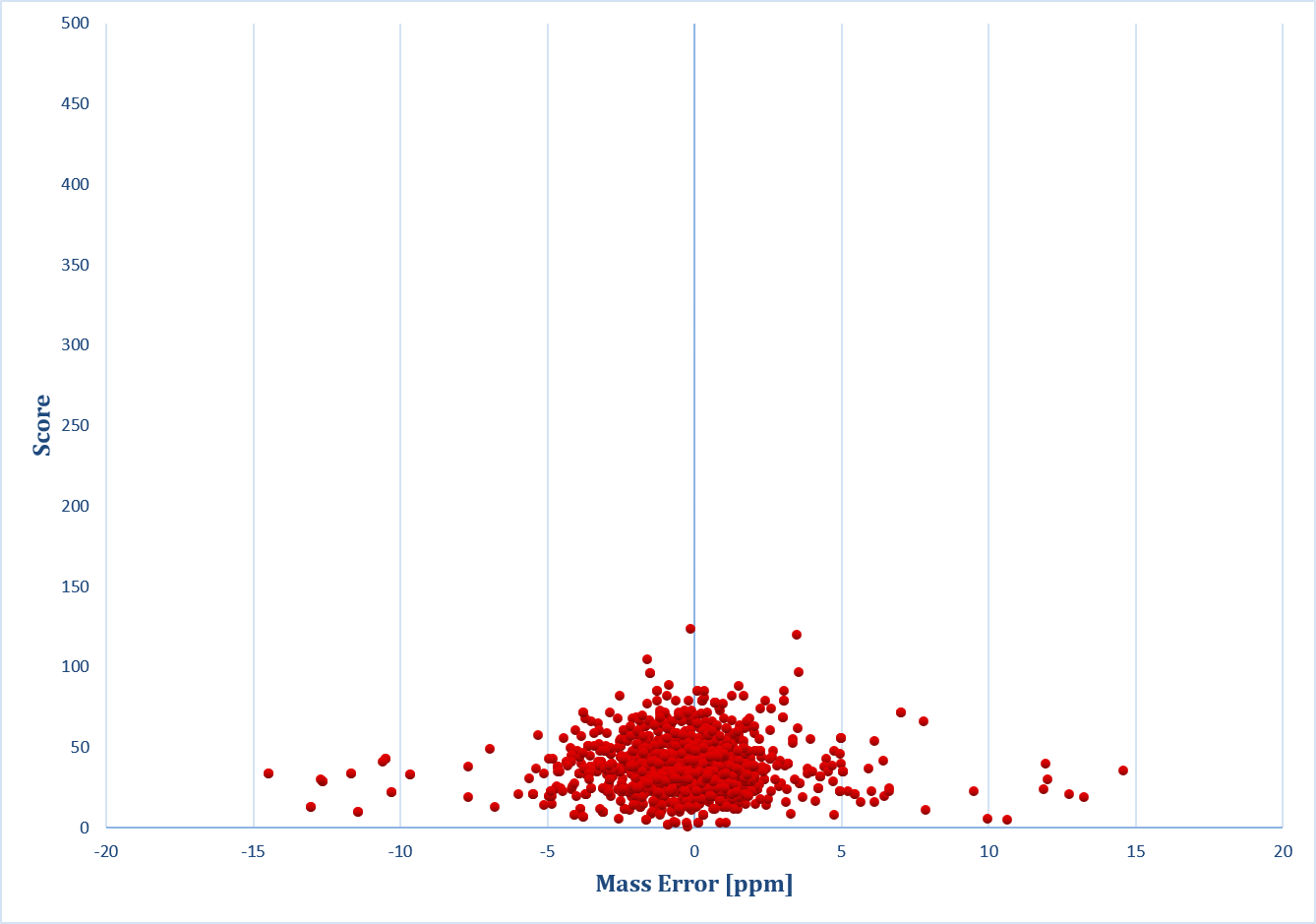

Supplement: Supplementary file 1 [file life-13-00765-s001.zip › Figure S1 Peptide matching error distribution.png]

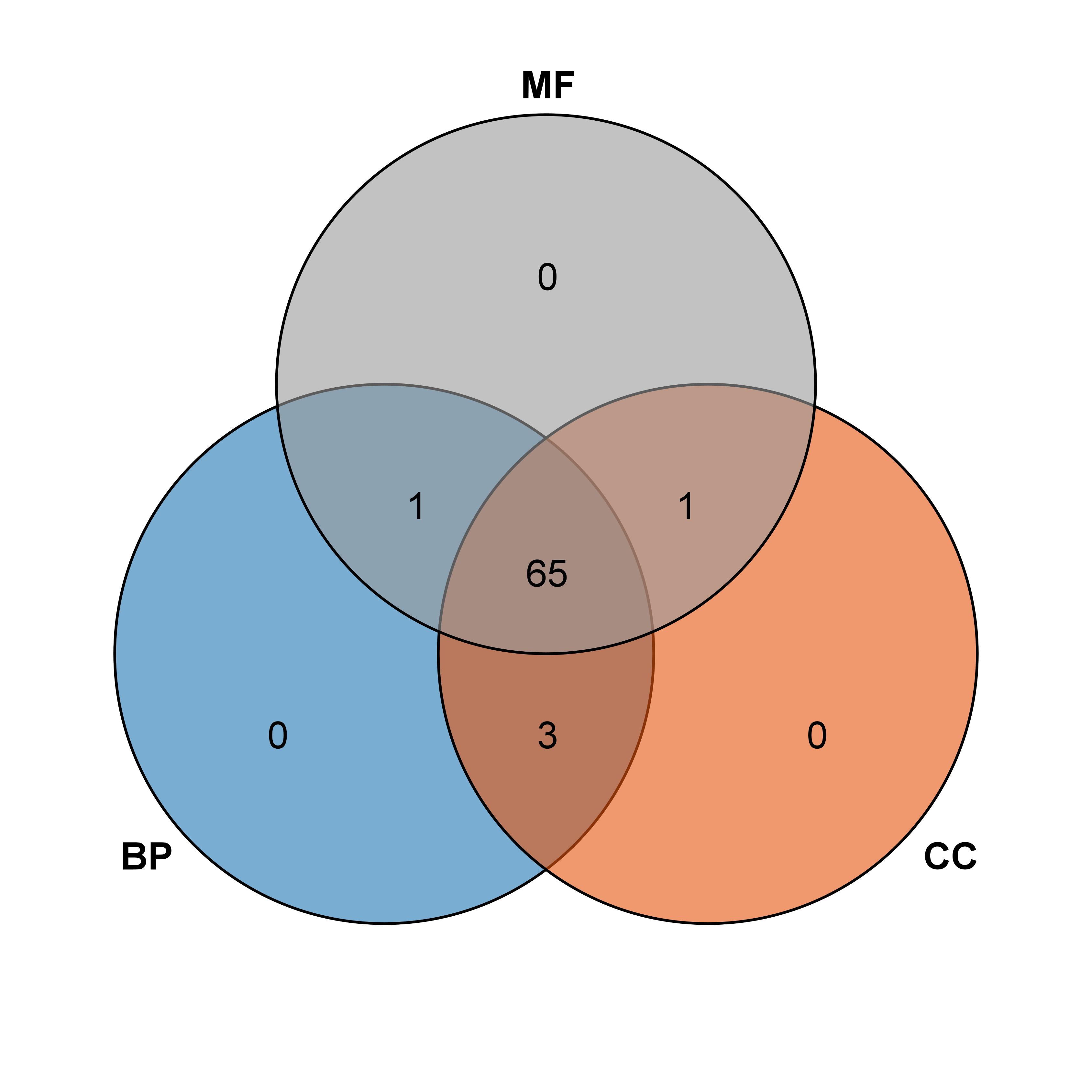

Supplement: Supplementary file 1 [file life-13-00765-s001.zip › Figure S2 Venn diagram for DEPs between FLZ and LZ group. BP Go biological process categories. CC GO cell component categories. MF GO molecular function categories..jpg]

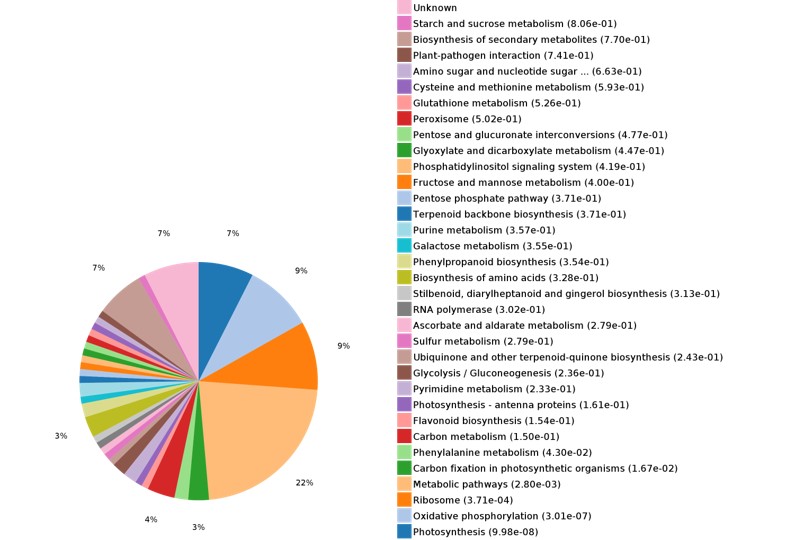

Supplement: Supplementary file 1 [file life-13-00765-s001.zip › Figure S3 KEGG enrichment of DEPs between FLZ and LZ group.jpg]
